# Early (≤10 Days) vs. Late (>10 Days) Tracheostomy in the Intensive Care Unit: Impact on Discontinuation of Sedation and Mechanical Ventilation

**DOI:** 10.3390/life16060891

**Published:** 2026-05-26

**Authors:** Angelo Buglione, Carmine Colella, Elena Pepe, Luca Gregorio Giaccari, Maria Caterina Pace, Vincenzo Pota, Dario Gaetano, Modestino Matarazzo, Pasquale Sansone

**Affiliations:** 1 U.O.C. Anesthesia and Intensive Care, AORN San Giuseppe Moscati, 83100 Avellino, Italy; anbuglione@gmail.com (A.B.); carminecolellamail@gmail.com (C.C.); dottelenapepe@gmail.com (E.P.); modestinomatarazzo75@gmail.com (M.M.); 2 Department of Woman, Child, General and Specialized Surgery, University of Campania “Luigi Vanvitelli”, 80138 Naples, Italy; mariacaterina.pace@unicampania.it (M.C.P.); vincenzo.pota@unicampania.it (V.P.); dario.gaetano1@studenti.unicampania.it (D.G.); pasquale.sansone@unicampania.it (P.S.)

**Keywords:** tracheostomy, intensive care unit, mechanical ventilation, sedation, observational study, timing

## Abstract

Background: The timing of tracheostomy in the intensive care unit (ICU) is debated because of its potential effects on comfort, sedation management, and ventilator weaning. Objective: To compare early (≤10 days) versus late (>10 days) tracheostomy with respect to discontinuation of sedation and invasive ventilation. Methods: Single-centre retrospective observational study. We included 52 consecutive ICU patients who underwent tracheostomy (January 2023–June 2025): 16 early and 36 late. Switching to dexmedetomidine was considered discontinuation of hypnotic sedation; transition to home mechanical ventilation (VAM) was considered discontinuation of invasive ventilation. Results: Sedation discontinuation occurred in 15/16 (93.8%) early vs. 35/36 (97.2%) late patients (*p* = 0.525). Discontinuation of invasive ventilation occurred in 12/16 (75.0%) early vs. 31/36 (86.1%) late patients (*p* = 0.431). Tracheostomy-to-sedation stop time: median 3 days [IQR 1–10] (overlapping between groups). Tracheostomy-to-ventilation stop time: median 17 days [IQR 10–27] (17 [11–33] early vs. 17 [10–25] late). ICU mortality: 3/16 (18.8%) vs. 6/36 (16.7%) (*p* = 1.00). Conclusions: In this retrospective cohort, no statistically significant differences emerged between early and late tracheostomy regarding discontinuation of sedation or invasive ventilation. However, given the retrospective design and small sample size, the study may have been underpowered to detect smaller but clinically relevant differences between groups. Prospective studies with larger sample sizes and severity-related variables may clarify any effects of timing.

## 1. Introduction

Tracheostomy is commonly used in the ICU for patients who need extended mechanical ventilation [1]. Potential advantages of tracheostomy compared with prolonged orotracheal intubation include greater patient comfort and a reduced need for sedation, facilitating faster weaning (discontinuation from ventilation), as well as a reduction in the incidence of nosocomial pneumonia and length of stay [1]. Indeed, performing a tracheostomy may improve respiratory mechanics and bronchial toileting, allow for early oral feeding, and reduce trauma from prolonged intubation to the larynx and trachea [2]. Consequently, once a tracheostomy has been performed, a marked decrease in the requirement for intravenous sedative and analgesic drugs is often observed [3]. In an observational study, Nieszkowska et al. documented that after tracheostomy the daily administration of opioids and benzodiazepines decreases by more than 10-fold, and the time spent in deep sedation falls from a median of 7 h to 1 h per day, without an increase in agitation [3]. These data confirm the clinical perception that tracheostomy enables lighter sedation management and earlier recovery of autonomy (mobilisation, feeding, and communication) in ventilated patients [3]. These benefits may be partly explained by a reduction in anatomical dead space and airway resistance, improved patient comfort, easier bronchial toileting, and a consequent decrease in the work of breathing.

Despite the potential benefits, the optimal timing for tracheostomy in the intensive care unit remains debated [2]. Historically, guidelines suggested delaying tracheostomy by approximately 3 weeks: in 1989, a U.S. consensus conference recommended proceeding with tracheostomy no earlier than 21 days of intubation in patients requiring prolonged mechanical ventilation [4]. However, subsequent studies have questioned this wait-and-see approach, exploring the hypothesis that earlier tracheostomy may improve clinical outcomes [4]. Numerous randomised clinical trials and meta-analyses have compared an “early” tracheostomy (variably defined, for example within 4 to 7 days from intubation) with a “late” or deferred tracheostomy (beyond 10 to 14 days, or performed only if necessary) in ventilated patients. However, the results of these studies have been discordant.

On the one hand, some small studies suggested marked benefits with early tracheostomy. Rumbak et al. (2004), in a randomised trial in medical patients, found that performing tracheostomy within 48 h of intubation compared with waiting ~2 weeks led to a significant reduction in mortality (31.7% vs. 61.7%) and pneumonia incidence (5% vs. 25%), as well as halving the duration of mechanical ventilation (approximately 7.6 vs. 17.4 days) and ICU length of stay [5]. These dramatic results fuelled the hypothesis that very early timing could substantially improve prognosis. Subsequently, meta-analyses have consolidated part of these data: for example, Hosokawa et al. (2015) pooled 12 randomised trials (2689 patients), showing that early tracheostomy is associated with more ventilator-free days (+2.12 days), a shorter duration of sedation (mean −5.1 days), and a shorter ICU stay (−5.1 days) compared with delayed tracheostomy, as well as a slight reduction in long-term mortality [6]. Another more recent systematic review (19 RCTs included) confirmed that earlier tracheostomy is associated on average with a reduction in the duration of mechanical ventilation and ICU length of stay, with a favourable effect also on mortality (risk ratio ~0.85) [2]. In a 2024 observational study conducted in China, Luo et al. reported that, after propensity score adjustment, patients tracheostomised within 7 days had significantly more sedation-free days (median ~21.5 vs. 16.5 days) and ventilator-free days (20.5 vs. 10.5) in the first month compared with those tracheostomised after day 7 [4]. Moreover, the incidence of ventilator-associated pneumonia (VAP) was significantly lower in the early group (10% vs. 22%) [4]. Such evidence suggests that earlier tracheostomy might accelerate clinical recovery by reducing the need for sedation and infectious complications related to prolonged intubation.

On the other hand, larger prospective studies with rigorous designs have not detected such clear advantages in terms of “hard” outcomes (mortality, VAP) associated with early tracheostomy. The multicentre TracMan trial (2013), conducted on nearly 900 patients in the UK, found no significant difference in 30-day mortality or in the other main secondary outcomes between patients randomised to tracheostomy within 4 days of ICU admission versus deferred tracheostomy at ≥10 days (if still necessary) [1]. Similarly, one of the largest European studies, the Italian trial by Terragni et al. (JAMA 2010), compared tracheostomy within 6 to 8 days versus after 13 to 15 days across 12 ICUs: the primary outcome (incidence of VAP) was lower in the early group (14% vs. 21%), but without statistical significance (*p* = 0.07) [7]. In the same study, earlier tracheostomy showed favourable trends in ventilator-free and ICU-free days in the subsequent 4 weeks but did not improve survival [7]. These results led many centres not to radically change practice, often maintaining a cautious (“wait and see”) approach in which tracheostomy is planned around day 10–14 of ventilation if the patient does not show signs of imminent weaning [1]. This “intermediate” timing is also reflected in international surveys: on average, tracheostomy is performed about 10 days after intubation [1], although there is wide variability and a non-negligible proportion of patients undergo tracheostomy early within the first week [1].

In light of this heterogeneity of findings in the literature and the lack of definitive consensus, we conducted a single-centre retrospective study to compare clinical outcomes related to sedation and mechanical ventilation in patients undergoing early versus late tracheostomy in our ICU. Specifically, we chose a 10-day cut-off from ICU admission because it represents both a pragmatic threshold close to current practice in our centre (tracheostomy typically performed within the second week when indicated) and a timing consistent with international surveys showing that tracheostomy is commonly performed around this time point. The aim was to evaluate whether earlier tracheostomy (≤10 days) is associated, compared with delayed tracheostomy (>10 days), with more frequent and/or faster discontinuation of both pharmacological sedation and mechanical ventilation. We also contextualised our results with respect to published evidence, including both international studies and relevant Italian contributions, including key historical reference works, to provide a comprehensive overview of the topic.

## 2. Materials and Methods

Study design and setting. Our study is a single-centre retrospective observational study conducted at the U.O.C. Anaesthesia and Intensive Care Unit of the AORN “San Giuseppe Moscati” (Avellino, Italy). This report follows the STROBE (Strengthening the Reporting of Observational Studies in Epidemiology) statement for observational studies.

During the study period, no major institutional changes in sedation or ventilatory weaning protocols were introduced.

Study period: We considered patients admitted to the ICU between January 2023 and June 2025 who underwent tracheostomy during their ICU stay.

Participants: Consecutive patients who underwent tracheostomy in the ICU during the study period were included. Exclusion criteria were age < 18 years and lack of the minimum required data (ICU admission and tracheostomy dates).

Operational definitions: ICU admission→tracheostomy time was calculated as the difference (in days) between the date of ICU admission and the date the tracheostomy was performed. Patients were classified into two groups according to the prespecified cut-off: early tracheostomy (≤10 days) versus late tracheostomy (>10 days).

Discontinuation of sedation: Event defined as interruption of continuous sedation and/or transition to analgesia-only management; for consistency, switching to dexmedetomidine (Dexdor^®^) was considered discontinuation of continuous hypnotic sedation. Dexmedetomidine was considered separately from continuous hypnotic sedation because it allows for a lighter, cooperative form of sedation, with less respiratory depression and greater patient interaction compared with traditional hypnotic agents.

Discontinuation of ventilation: Event defined as disconnection from invasive mechanical ventilation. Transition to home-assisted/non-invasive ventilation (home VAM) was considered discontinuation of invasive ventilation for the purpose of the outcome.

Outcomes: Primary outcomes: (1) Proportion of patients with sedation discontinuation. (2) Proportion of patients with discontinuation of invasive ventilation. Secondary outcomes: Discharge alive from the ICU, “not deceased” outcome (discharged alive or transferred), and ICU mortality.

Data collection and pseudonymisation: Data were retrospectively extracted from clinical documentation (electronic clinical records) and recorded in a dedicated CRF. To ensure confidentiality, each patient was coded with a sequential identifier (P01, P02, …). The re-identification file was kept separate and accessible only to authorised personnel according to institutional procedures and Regulation (EU) 2016/679 (GDPR).

Handling of missing data: For dichotomous outcomes, patients who did not achieve discontinuation of sedation/ventilation before death or transfer were classified as “event not achieved”. For analyses of tracheostomy→event times, times were calculated only among patients who actually achieved the event (“Never discontinued” cases were excluded from the calculation of medians).

Statistical analysis: Categorical variables are described as n (%). Differences in proportions between groups were tested using a two-sided Fisher’s exact test, appropriate in the presence of cells with low expected frequencies. The absolute risk difference (Risk Difference, RD) between groups was also estimated with a 95% confidence interval (CI) using Newcombe’s (score) method. Time variables are reported as median [interquartile range, IQR]. Statistical significance was set at α = 0.05 (two-sided).

Software: Statistical analyses and figures were generated in Python (v3.10) using standard libraries for data analysis and statistical testing (e.g., Pandas/Numpy and SciPy; Fisher’s exact test) and graphical libraries (Matplotlib version 3.10.8, Seaborn version 0.13.1). Preliminary data handling was performed in a spreadsheet environment (Excel).

Ethical aspects: The protocol was submitted for review to the Campania 3 Ethics Committee (approval number 218/2026); given the retrospective nature and the use of pseudonymised data, a waiver of informed consent was requested, in accordance with current regulations and institutional procedures.

## 3. Results

Sample characteristics: A total of 52 consecutive patients (n = 52) who underwent tracheostomy in the ICU during the study period were included. Based on the prespecified timing criterion, 16 patients (30.8%) received an early tracheostomy (within 10 days) from admission, whereas 36 patients (69.2%) received a late tracheostomy (after 10 days). The unequal distribution between the early and late tracheostomy groups should be considered when interpreting the results, as it may have reduced the ability to detect statistically significant between-group differences. Table 1 reports the general characteristics of the two groups.

Overall, 37/52 (71.2%) were male and 15/52 (28.8%) were female; sex distribution was similar between groups (early 75.0% vs. late 69.4%). By definition, ICU admission→tracheostomy time differed in median: 7 days [IQR 5–8] in the early group versus 17 days [IQR 14–23] in the late group (overall median 14 days [9–20]). Other potentially relevant baseline clinical variables, including severity scores (e.g., APACHE II or SOFA), indication for tracheostomy, neurological status, cause of respiratory failure, and comorbidities, were not uniformly available across all cases and therefore could not be included in this preliminary analysis.

Primary clinical outcomes—proportions: Table 2 summarises the primary dichotomous outcomes in the two groups (percentages of patients who achieved discontinuation of sedation and ventilation, in addition to some secondary survival outcomes).

**Figure 1:**
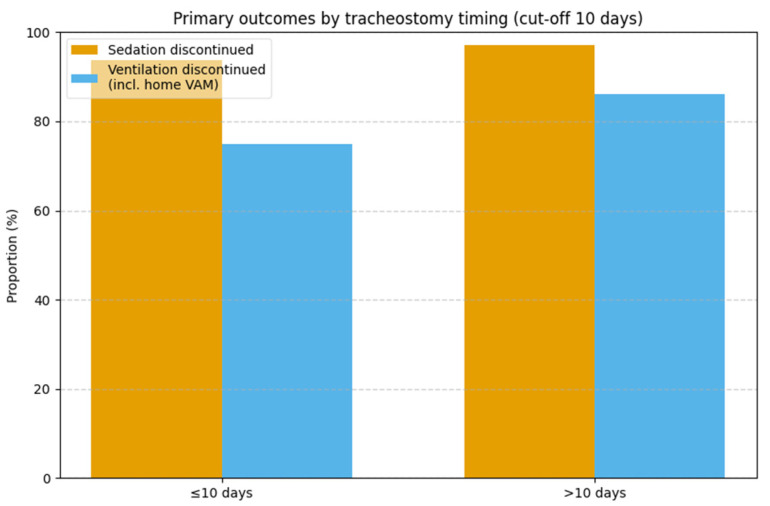
Proportion of patients with discontinuation of sedation and ventilation.

In almost all patients it was possible to discontinue continuous sedation before ICU discharge: 15/16 (93.8%) in the early group versus 35/36 (97.2%) in the late group, with no significant difference (*p* = 0.525, Fisher’s exact test). The proportion of patients weaned from invasive ventilation (including those transitioned to home VAM) was 12/16 (75.0%) in the early group versus 31/36 (86.1%) in the late group (*p* = 0.431). The absolute risk difference for “ventilation discontinued” was −11.1% (95% CI −36.8%; +9.8%), compatible with the absence of certain differences. Regarding secondary outcomes, ICU mortality was 18.8% (3/16) in the early group versus 16.7% (6/36) in the late group (*p* = 1.00). The proportions of the main outcomes in the two groups are also summarised in Figure 1.

Time from procedure to outcomes: Table 3 presents the times (median and IQR, in days) from tracheostomy to achievement of clinical outcomes.

**Figure 2:**
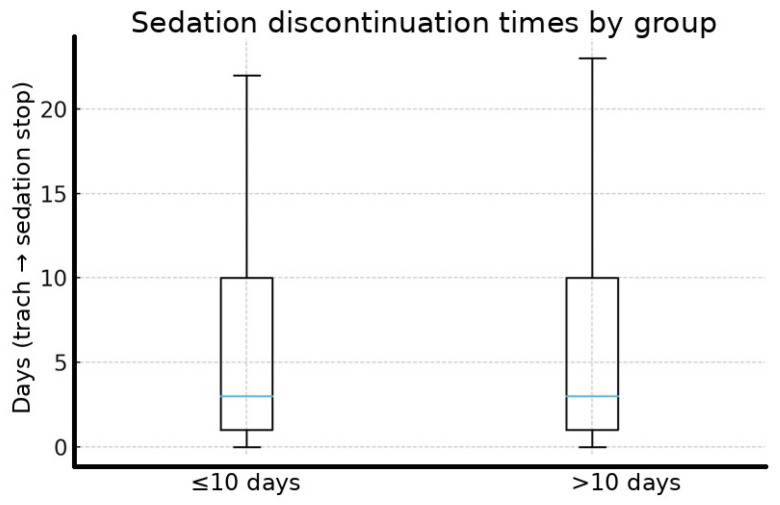
Sedation duration in the two groups (boxplot).

**Figure 3:**
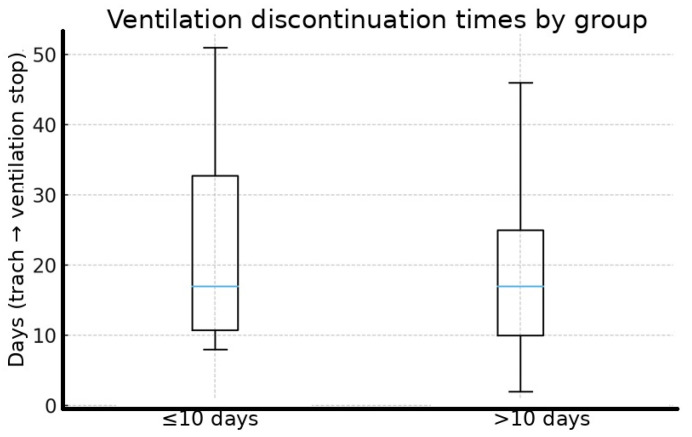
Ventilation duration in the two groups (boxplot).

Among patients who discontinued sedation, the median tracheostomy→sedation stop time was 3 days [IQR 1–10] overall (n = 42) and was comparable between groups: 3 [1–10] days both in the early group (n = 14) and in the late group (n = 28). Regarding tracheostomy→discontinuation of invasive ventilation, among patients in whom weaning occurred the median was 17 days [IQR 10–27] (n = 43). Again, no relevant differences were observed: 17 days [IQR 11–33] in the early group (n = 12) versus 17 days [IQR 10–25] in the late group (n = 31). Time distributions are illustrated in Figure 2 (sedation) and Figure 3 (ventilation).

The identical median times observed in the two groups for both sedation discontinuation and ventilation discontinuation are noteworthy. This finding suggests that, in our cohort, post-tracheostomy clinical trajectories may have been influenced more by patient condition, local sedation and weaning practices, or readiness for recovery at the time of the procedure than by tracheostomy timing alone.

2 × 2 statistical comparisons: Table 4 reports contingency tables, *p*-values (two-sided Fisher), and absolute risk differences (RD) with 95% CIs. For sedation discontinuation: *p* = 0.525; RD −3.5% (95% CI −25.7%; +9.0%). For discontinuation of invasive ventilation: *p* = 0.431; RD −11.1% (95% CI −36.8%; +9.8%). Secondary outcomes (discharge alive and ICU mortality) showed no significant differences between groups.

## 4. Discussion

Summary of the main findings: In this retrospective cohort of critically ill patients undergoing tracheostomy, we found no significant differences related to the timing of the procedure (early within 10 days vs. late after 10 days) in relation to the outcomes of sedation discontinuation and weaning from mechanical ventilation. Virtually all patients in both groups were able—consistent with disease severity—to stop continuous sedation and be liberated from the ventilator before discharge or transfer. The median timing of these events, measured in days from tracheostomy, overlapped in the two populations. Specifically, the post-tracheostomy time to discontinue sedative drugs (approximately 3 days on average) and the time to complete separation from the ventilator (approximately 2 weeks) were identical in both groups. Moreover, the proportions of patients who actually achieved these clinical targets did not differ significantly (93–97% for sedation, 75–86% for respiratory weaning). Overall, therefore, our experience does not show clinical advantages nor disadvantages associated with performing tracheostomy within 10 days compared with a more delayed procedure, at least with regard to sedation and mechanical ventilation management. The slightly lower success proportions observed in the early tracheostomy group may reflect unmeasured differences in baseline clinical severity. Patients selected for earlier tracheostomy may have had a worse predicted ventilatory prognosis or greater clinical complexity, which could have attenuated any potential benefit of earlier intervention. This possibility should be considered when interpreting the absence of statistically significant differences.

Comparison with the literature: Our results fall within the area populated by conflicting evidence on the optimal timing of tracheostomy in intensive care. On the one hand, they confirm what has emerged from the largest randomised trials conducted in this field, which generally did not document radical differences in major outcomes between early and late tracheostomy. For instance, the TracMan study (UK)—which defined early as within 4 days and late as after 10 days—reported no benefit of the earlier approach either in terms of 30-day mortality or secondary endpoints such as ICU length of stay or major complications [1]. Similarly, in our sample, choosing to perform tracheostomy within the second week did not improve the likelihood of weaning nor shorten post-procedure timelines compared with a more wait-and-see approach. The Italian trial by Terragni et al. (early at 7 days vs. late at 14 days) reached comparable conclusions, finding no significant differences in the incidence of ventilator-associated pneumonia (VAP)—the primary outcome—between the two groups [7]. In that study there was a trend in favour of early tracheostomy (14% vs. 21% VAP), but not statistically reliable (*p* = 0.07), consistent with the lack of significance observed in our analysis for sedation and weaning endpoints. It is noteworthy that Terragni et al. nonetheless observed some advantage in parameters such as ventilator-free and ICU-free days within the first 4 weeks (hazard ratio ~0.70 in favour of the early group) [7], suggesting that a moderate benefit may exist but be difficult to detect without large case series. In our dataset, although numbers are too small for robust calculations, not even a qualitative trend favouring early tracheostomy emerged—indeed, success proportions (sedation/ventilation discontinued) were slightly lower in the early group, likely reflecting unmeasured differences in clinical severity.

On the other hand, the literature provides substantial evidence supporting benefits of earlier tracheostomy, particularly regarding duration of ventilation, sedative use, and respiratory infections. Several smaller randomised studies and meta-analyses suggest advantages when tracheostomy is performed within the first week [8,9,10,11,12,13,14,15,16,17,18,19]. For example, the meta-analysis by Siempos et al. (2015) showed that early tracheostomy (within 7 days) tends to be associated with a reduced incidence of VAP compared with late tracheostomy or no tracheostomy (odds ratio ~0.60 in favour of early) [8]. In our study we did not specifically assess pneumonia incidence; however, the proportion of chronically ventilated (not weaned) patients was slightly higher in the early group, which might have translated into a similar or higher infectious risk (hypothetically, more severe patients requiring early tracheostomy could also be more susceptible to VAP, offsetting potential benefits). Previous evidence also indicates that tracheostomy itself may reduce sedation requirements and improve patient comfort and interaction [3,9]. One might therefore expect that earlier tracheostomy would save sedation days. Indeed, some comparative studies have shown this: for instance, Liu et al. (2015) [11] and Wang et al. (2011) [14], in their respective pooled analyses of trials, reported that early tracheostomy is associated with about 5 fewer days of sedation compared with late tracheostomy [6,10,11,12,13,14,15,16]. Nieszkowska et al. (2005), although without a time-matched control group, documented a drastic decrease in sedation after tracheostomy, indicating that the procedure is a turning point in sedative management [3]. In contrast, our analysis shows no difference in residual post-tracheostomy sedation time between early and late procedures. One possible explanation is that both groups, at the time of tracheostomy, were ready for sedation lightening: in the early group, tracheostomy was likely decided when the patient was reaching criteria to reduce sedation (for example, to begin weaning); in the late group, while waiting for tracheostomy clinicians may have progressively reduced sedation already in the days preceding the procedure. In other words, beyond the endotracheal tube itself, good sedation management (with daily awakening protocols, frequent reassessment of sedative needs, etc.) can lead to reduction of sedative drugs as soon as clinical conditions allow—regardless of whether tracheostomy has already been performed. It has been reported that sedation requirements often decrease markedly around day 7–10 of ventilation in standard weaning approaches, irrespective of tracheostomy. This might explain why in our cohort sedation discontinuation times were identical: late-group patients had already begun to “free themselves” from sedation even before being tracheostomised. Some authors have suggested that a policy of light sedation and daily monitoring of agitation can minimise the additional benefit of early tracheostomy on sedative consumption [9,20]. Moreover, our “sedation discontinued” endpoint included transition to low-dose dexmedetomidine, which effectively allows for patients to remain awake and cooperative: it is plausible that many late-group patients received less sedation already before tracheostomy, perhaps with protective ventilation and cooperation, thereby reducing any difference compared with those who underwent early tracheostomy.

Another aspect to consider is the temporal cut-off adopted across different studies. In our work we defined “early” as within 10 days, meaning that patients tracheostomised on day 9 or 10 (who in the literature would sometimes be classified as “late” in studies using a 7-day threshold) were included in our early group. This may have attenuated differences: many of our “early” cases were not truly very early tracheostomies (median 7 days, with IQR 5–7, thus few performed before day 5). Conversely, the >10-day threshold meant that the late group included patients tracheostomised in the third–fourth week (median 17 days). Thus, we are broadly comparing a “within the second week” approach versus “after the second week.” In the literature, marked differences in outcomes have been observed mainly when comparing a very early strategy (within the first 4–5 days) with a very late strategy (≥2 weeks) [6]. It is possible that in our centre, tracheostomy is rarely performed in the very first days unless under highly selected conditions; therefore, our comparison lacks the ultra-early extreme that elsewhere has shown benefits (e.g., Rumbak 2004 with tracheostomy at 2 days). This may have contributed to the absence of differences: in practice, our “early” group might be considered an intermediate timing compared with other trials. This consideration highlights the importance of a shared standard in definitions of early versus late tracheostomy, which vary in the literature (within 4, 7, or 10 days depending on authors) [2].

Study limitations: These results should be interpreted in light of several important methodological limitations. First, this is a retrospective, single-centre study with a small sample size (N = 52): statistical power is limited and does not allow for complete exclusion of clinically relevant differences, particularly for moderate effects. Moreover, the imbalance between the early and late tracheostomy groups (16 vs. 36 patients) further reduced the statistical power of between-group comparisons. Therefore, the absence of statistically significant differences should not be interpreted as evidence of equivalence between the two strategies. A post hoc assessment suggests we would have been able to detect statistical significance only for fairly large effects (e.g., differences > 20–30% in proportions), whereas subtler effects may have been missed. Second, we did not perform an adjusted multivariable analysis: the comparison between groups is not randomised and is therefore subject to potential selection bias. Indeed, the timing of tracheostomy was likely influenced by physician judgment, expected ventilatory prognosis, neurological condition, and overall clinical severity. Consequently, causality cannot be inferred from the observed associations. Patients undergoing earlier tracheostomy may have represented a clinically more severe subgroup, potentially introducing indication bias and confounding by severity. It is plausible that patients receiving early tracheostomy had different characteristics (for example, greater severity or a worse predicted ventilatory prognosis) compared with those receiving late tracheostomy—precisely because the decision to perform tracheostomy within the first week is often made when prolonged ventilation is anticipated [1]. In our dataset we did not identify obvious demographic imbalances, but variables such as severity scores (e.g., APACHE II) or primary diagnosis were not reported and could have influenced results. Similarly, neurological status, indication for tracheostomy, cause of respiratory failure, and comorbidity burden were not systematically available. The absence of these variables limits comparability between groups and prevents identification of independent predictors associated with successful sedation discontinuation or ventilatory weaning. For instance, ICU mortality in the early group was slightly higher (18.8% vs. 16.7%) and the proportion of patients not weanable also higher (25% vs. 13.9%), suggesting that early-group patients might have been more critically ill at baseline (although the small sample does not allow for confirmation). In that case, the lack of differences could imply that early tracheostomy compensated for a worse prognosis, leading to outcomes similar to the less severe group (a speculative hypothesis). Only adjusted analyses (e.g., matching or multivariable models) could clarify this point, but a larger sample would be required. Another limitation is the lack of assessment of other potentially relevant outcomes: for example, VAP incidence, total ventilation days, total ICU length of stay, tracheostomy-related complications, or long-term outcomes (in-hospital mortality, total hospitalisation duration). Our focus was deliberately targeted on sedation and weaning, but this does not provide a complete picture of the effect of tracheostomy timing. For example, it is possible that in our cohort, early tracheostomy yielded benefits in terms of fewer laryngotracheal complications or improved comfort (not objectively measured), aspects we did not capture. Additionally, we have no data on total sedation burden (cumulative drug doses) or sedation/agitation scores in the two groups: we limited ourselves to a dichotomous endpoint (sedation yes/no). More detailed studies should include more granular measures, such as sedation days saved or daily doses of sedatives and analgesics, to determine whether differences emerge. It should also be noted that the very definition of “sedation discontinued” in our study is peculiar (including dexmedetomidine as the end of sedation). Others may define this endpoint differently, making results less comparable across series. Similarly, we considered “ventilation discontinued” to include transition to home non-invasive ventilation: this was because our clinical aim was to verify whether tracheostomy allowed for liberation of the natural airway and restoration of autonomous breathing capacity (albeit with non-invasive support). In different contexts this might not be the primary outcome (many studies consider ventilator-free days in ICU, etc.). Finally, the single-centre design limits the generalisability of our findings. Sedation and weaning protocols may vary substantially between institutions, and results observed in our ICU may not be fully reproducible in centres with different clinical practices. As noted, particularly attentive sedation management may have mitigated differences; elsewhere, with different protocols, results could differ. For example, if a centre tends to keep patients deeply sedated as long as they are intubated, then early tracheostomy in that setting would have a more marked impact on sedation reduction (because pre-tracheostomy sedation would remain high). In our centre, however, the tendency is already to reduce sedation early even with an endotracheal tube whenever possible, which may explain why early tracheostomy added little in terms of further sedation reduction. These differences in clinical practice are difficult to standardise but should be considered when interpreting the various studies [21].

Clinical implications: In light of our results, in a context similar to ours (a general ICU with established protocols), a tracheostomy performed around 7–10 days after intubation may not produce tangible improvements either in shortening time to sedative discontinuation or in promoting earlier respiratory weaning compared with a tracheostomy performed in the third week. This suggests that timing decisions should remain individualised, taking into account other factors in particular: neurological prognosis, expected recovery, prevention of damage from prolonged intubation (e.g., accidental extubations, tracheal injuries), the need to free ICU beds, etc. Potential advantages of early tracheostomy—such as improved comfort and possibly fewer pneumonias—must be balanced against procedural risks and uncertainty regarding benefits on hard outcomes. Our findings do not support a clear advantage of performing tracheostomy before day 10 in this specific cohort; however, the exploratory nature of the study prevents definitive clinical recommendations. This aligns with some guidelines suggesting waiting ~10–14 days to determine whether the patient will recover without tracheostomy, unless there are clear indications to perform it earlier (such as inability to wean despite improvement, or the need for long-term airway protection, etc.). On the other hand, the fact that many studies report benefits with tracheostomy within the first week implies that, in selected patient subgroups (e.g., younger patients without severe neurological damage, with a high likelihood of respiratory recovery once liberated early from sedation), a more aggressive approach might reduce ventilation duration [5,6]. This remains a grey area requiring further research.

Generalisability and next steps: Despite the limitations described, this study demonstrates the feasibility of a pragmatic retrospective approach to evaluate the impact of tracheostomy timing on outcomes. The simple criteria and clinical endpoints used could be readily adopted in a multicentre setting or larger cohorts. A future initiative could extend data collection to other Italian centres, increasing sample size and allowing for adjusted or stratified analyses (for example, distinguishing neurological vs. non-neurological patients, COVID-19 vs. non-COVID-19, etc.). Moreover, it would be of interest to include additional variables: for example, quantifying cumulative propofol/midazolam/fentanyl use in the two groups, assessing 28-day ventilator-free days, and measuring recovery quality (e.g., how many patients are able to speak or feed orally within a given number of days after tracheostomy). Another avenue would be to analyse long-term outcomes: our analysis stops at ICU discharge, but we do not know whether earlier or later tracheostomy influenced subsequent ward course or overall in-hospital mortality. Some evidence suggests a possible impact on 1-year mortality (Hosokawa found slightly lower long-term mortality with early tracheostomy) [6], but this remains controversial. Finally, with the experience of the COVID-19 pandemic, tracheostomy timing has become even more critical: dedicated studies in patients with SARS-CoV-2 ARDS have explored different timeframes based on infectivity and severity. Integrating those data (for example, Italian studies on the role of tracheostomy in COVID-19 [2]) could further enrich the overall understanding.

A brief historical perspective is also relevant. The concept of surgically securing the airway has ancient roots, with early descriptions traditionally attributed to Greek physicians such as Asclepiades and later discussed by Galen [22]. Although modern tracheostomy techniques were developed many centuries later, these early references highlight the long-standing medical interest in restoring airway patency in life-threatening obstruction.

## 5. Conclusions

In conclusion, in our single-centre retrospective study, no statistically significant differences were identified regarding discontinuation of sedation and ventilatory weaning between patients undergoing early tracheostomy within 10 days of ICU admission and those undergoing late tracheostomy after 10 days. These results, consistent with those reported by some large clinical studies, suggest that in a setting characterised by homogeneous sedation and weaning protocols, the effect of tracheostomy timing on these specific outcomes may be marginal. The approach we propose—clearly distinguishing patients according to a prespecified temporal cut-off and evaluating outcomes—proved clear and reproducible and can be readily extended in future studies by enrolling a larger number of patients and including additional clinical variables for adjusted analyses. The question remains open as to whether there are patient subpopulations that may instead benefit significantly from earlier tracheostomy: only large multicentre prospective studies or updated meta-analyses will be able to definitively clarify the role of optimal tracheostomy timing in improving outcomes in critically ill patients. In the meantime, the decision on when to perform tracheostomy should be individualised, balancing potential benefits (comfort, reduced sedative use, prevention of complications from prolonged intubation) against risks, and taking into account the patient’s clinical course during the first two weeks of mechanical ventilation.

## Figures and Tables

**Table 1:** Patient characteristics in the two groups.

Characteristics	Early (≤10 Days)	Late (>10 Days)	Total
No.	16	36	52
Males n (%)	12 (75.0%)	25 (69.4%)	37 (71.2%)
Females n (%)	4 (25.0%)	11 (30.6%)	15 (28.8%)
ICU admission→tracheostomy (days, median [IQR])	7 [5–8]	17 [14–23]	14 [9–20]

**Table 2:** Main outcomes in the two groups.

Outcome	Early n (%)	Late n (%)
Sedation discontinued	15 (93.8%)	35 (97.2%)
Invasive ventilation discontinued (incl. home VAM)	12 (75.0%)	31 (86.1%)
Discharged alive	9 (56.2%)	26 (72.2%)
Not deceased (discharged + transferred)	13 (81.2%)	30 (83.3%)
Died	3 (18.8%)	6 (16.7%)

**Table 3:** Sedation and ventilation times (days).

Time (Days)	Early (≤10 Days)	Late (>10 Days)	Total
Trach→sedation stop, median [IQR] (n)	3 [1–10] (14)	3 [1–10] (28)	3 [1–10] (42)
Trach→ventilation stop, median [IQR] (n)	17 [11–33] (12)	17 [10–25] (31)	17 [10–27] (43)

**Table 4:** Contingency tables and statistical analysis (Fisher’s exact test, RD, 95% CI).

Endpoint	a (≤10 and Event)	b (≤10 and No)	c (>10 and Event)	d (>10 and No)	*p* (Two-Sided Fisher)	RD Early—Late (95% CI)
Sedation discontinued	15	1	35	1	0.525	−3.5% (−25.7%; +9.0%)
Invasive ventilation discontinued (incl. home VAM)	12	4	31	5	0.431	−11.1% (−36.8%; +9.8%)
Discharged alive	9	7	26	10	0.340	−16.0% (−41.9%; +10.3%)
Not deceased (discharged + transferred)	13	3	30	6	1.000	−2.1% (−27.9%; +17.4%)

Note: In some patients (Table S1), it was not possible to calculate tracheostomy→sedation stop and/or ventilation stop times because the event was not achieved before death or transfer. In the current dataset, tracheostomy→sedation stop time is available for 42/52 patients (14/16 early; 28/36 late) and tracheostomy→ventilation stop time for 43/52 (12/16 early; 31/36 late). Time medians are therefore calculated only among patients who achieved the event.

## Data Availability

The dataset contains pseudonymised clinical information and, for confidentiality reasons, is not publicly available; it may be made available upon reasonable request to the PI, subject to institutional/Ethics Committee authorisation.

## Supplementary Materials

The following supporting information can be downloaded at: https://www.mdpi.com/article/10.3390/life16060891/s1, Table S1: Patient-level details (anonymous ID code).

## Abbreviations

The following abbreviations are used in this manuscript:

ICU	Intensive Care Unit
VAM	ventilator
RD	absolute risk difference
CI	confidence interval
IQR	interquartile range
