# Supplementary material for: Early (≤10 Days) vs. Late (>10 Days) Tracheostomy in the Intensive Care Unit: Impact on Discontinuation of Sedation and Mechanical Ventilation
Source: Life (Basel). 2026 May 26;16(6):891. doi: 10.3390/life16060891 (PMC13301516; doi:10.3390/life16060891)
Supplement: Supplementary file 1 [file life-16-00891-s001.zip › life-4324621-supplementary.pdf]

Table S1. Patient-level details (anonymous ID code)

Patient ID	Sex	Admission	Tracheostomy	Sedation discontinued	Ventilation discontinued	Discharge/ Death	Final outcome
P01	M	09/10/2023	22/10/2023	20/11/2023	20/11/2023	28/11/2023	Discharged
P02	M	24/04/2024	28/04/2024	08/05/2024	Never discontinued	17/05/2024	Transferred
P03	F	24/09/2023	14/10/2023	03/10/2023	25/11/2023	13/12/2023	Discharged
P04	M	29/10/2023	03/11/2023	25/11/2023	30/11/2023	06/12/2023	Discharged
P05	M	28/09/2023	30/09/2023	Never discontinued	Never discontinued	15/10/2023	Died
P06	F	20/11/2023	04/12/2023	07/12/2023	27/12/2023	11/01/2024	Discharged
P07	M	01/12/2023	07/12/2023	23/12/2023	24/12/2023	10/01/2024	Discharged
P08	F	11/04/2024	09/05/2024	09/05/2024	Home VAM 20/05/2024	21/05/2024	Discharged
P09	F	06/04/2024	13/04/2024	07/04/2024	23/04/2024	18/05/2024	Discharged
P10	M	04/10/2023	19/10/2023	24/10/2023	30/11/2023	05/01/2024	Discharged
P11	M	29/06/2024	06/07/2024	14/07/2024	Never discontinued	21/07/2024	Died
P12	M	02/02/2024	19/02/2024	29/02/2024	03/03/2024	08/03/2024	Discharged
P13	F	17/05/2024	30/05/2024	31/05/2024	21/06/2024	21/06/2024	Discharged
P14	M	04/02/2024	24/02/2024	26/02/2024	Home VAM 05/03/2024	06/03/2024	Discharged
P15	M	06/05/2024	10/05/2024	11/05/2024	Home VAM 27/05/2024	28/05/2024	Discharged
P16	M	28/06/2024	08/07/2024	29/08/2024	28/08/2024	05/09/2024	Discharged
P17	M	22/04/2024	09/05/2024	06/05/2024	18/05/2024	17/06/2024	Discharged
P18	M	26/05/2024	31/05/2024	02/06/2024	Home VAM 17/07/2024	18/07/2024	Discharged
P19	M	08/06/2024	20/06/2024	14/06/2024	Never discontinued	17/06/2024	Died
P20	M	29/03/2024	05/04/2024	06/04/2024	16/04/2024	22/04/2024	Transferred
P21	M	24/03/2024	05/04/2024	21/04/2024	22/04/2024	24/04/2024	Transferred
P22	F	10/04/2024	28/04/2024	01/07/2024	18/05/2024	05/07/2024	Discharged
P23	M	21/07/2023	29/08/2023	21/09/2023	Home VAM 21/09/2023	22/09/2023	Discharged
P24	M	30/04/2024	22/05/2024	23/05/2024	25/05/2024	28/06/2024	Discharged
P25	M	05/06/2024	27/06/2024	17/07/2024	08/07/2024	01/08/2024	Discharged
P26	M	25/04/2023	18/05/2023	04/05/2023	Never discontinued	06/06/2023	Died
P27	M	12/04/2023	03/05/2023	16/04/2023	Home VAM 22/05/2023	29/06/2023	Discharged
P28	F	11/01/2024	25/01/2024	26/01/2024	24/02/2024	09/04/2024	Died
P29	M	16/09/2024	14/10/2024	26/10/2024	28/10/2024	03/01/2025	Discharged
P30	F	28/08/2024	13/09/2024	17/09/2024	08/10/2024	15/10/2024	Discharged
P31	F	29/12/2023	25/01/2024	01/02/2024	03/02/2024	20/03/2024	Discharged
P32	F	27/08/2024	13/09/2024	20/09/2024	Never discontinued	16/10/2024	Died
P33	M	29/09/2024	23/10/2024	Never discontinued	Never discontinued	06/11/2024	Died
P34	M	07/11/2024	02/12/2024	04/12/2024	Home VAM 15/01/2025	17/01/2025	Transferred
P35	M	15/12/2024	20/12/2024	30/12/2024	Never discontinued	04/01/2025	Died
P36	M	06/12/2024	23/12/2024	27/12/2024	01/01/2025	16/01/2025	Transferred
P37	M	14/02/2025	27/02/2025	28/02/2025	Home VAM 24/03/2025	24/03/2025	Discharged
P38	F	21/02/2025	28/02/2025	04/03/2025	Home VAM 08/03/2025	10/03/2025	Transferred
P39	M	22/03/2025	31/03/2025	01/04/2025	15/04/2025	08/05/2025	Transferred
P40	M	03/05/2025	15/05/2025	10/05/2025	Home VAM 10/06/2025	10/06/2025	Transferred
P41	F	11/08/2024	24/08/2024	26/08/2024	14/09/2024	17/09/2024	Discharged
P42	M	16/08/2024	31/08/2024	02/09/2024	10/09/2024	26/09/2024	Discharged
P43	F	22/08/2024	31/08/2024	31/08/2024	Home VAM	11/09/2024	Discharged

					10/09/2024		
P44	M	11/03/2024	03/04/2024	20/03/2024	Home VAM 16/04/2024	17/04/2024	Discharged
P45	M	27/03/2024	15/04/2024	25/04/2024	17/04/2024	07/05/2024	Discharged
P46	M	08/07/2024	02/08/2024	02/08/2024	06/08/2024	01/10/2024	Discharged
P47	M	02/09/2023	13/09/2023	16/09/2023	23/09/2023	17/10/2023	Discharged
P48	M	08/12/2023	22/12/2023	23/12/2023	Home VAM 02/01/2024	03/01/2024	Discharged
P49	F	03/03/2024	15/03/2024	18/03/2024	Home VAM 30/04/2024	03/05/2024	Discharged
P50	M	11/06/2023	04/07/2023	05/07/2023	Never discontinued	06/07/2023	Died
P51	M	03/07/2023	13/07/2023	15/07/2023	10/08/2023	24/08/2023	Discharged
P52	F	19/06/2023	26/06/2023	27/06/2023	Home VAM 12/08/2023	24/08/2023	Discharged
